# Effective Anti-Oxidation Repair Coating for C/C Brake Materials Comprising Lead-Borosilicate and Bismuth-Borosilicate Glass

**DOI:** 10.3390/ma15082827

**Published:** 2022-04-12

**Authors:** Mengjia Deng, Xiaoyu Xia, Juanli Deng, Kaiyue Hu, Chenghua Luan, Xu Ma, Shangwu Fan, Peng Wang

**Affiliations:** 1 School of Materials Science and Engineering, Chang’an University, Xi’an 710064, China; 2019231003@chd.edu.cn (M.D.); 2019131043@chd.edu.cn (X.X.); 2017131011@chd.edu.cn (K.H.); 2 Department of Chemistry, Materials and Chemical Engineering “Giulio Natta”, Politecnico di Milano, P.za Leonardo Da Vinci 32, 20133 Milano, Italy; 3 No. 52 Institute of China North Industry Group, Yantai 264003, China; g115385817@163.com; 4 Science and Technology on Thermostructural Composite Materials Laboratory, Northwestern Polytechnical University, Xi’an 710072, China; m_xu@mail.nwpu.edu.cn; 5 Xi’an Xinyao Ceramic Composite Material Co., Ltd., Xi’an 710199, China; pengwangmac@163.com

**Keywords:** borosilicate glass, lead glass, bismuth glass, antioxidation coating, C/C composites

## Abstract

To achieve effective antioxidation of on-site repair coating for C/C brake materials in the full temperature range (500–900 °C), lead glass and bismuth glass were introduced into the borosilicate glass to acquire the protective coatings. Before preparing coating samples, the thermal gravity characteristics of the lead/bismuth–borosilicate glass powders were analyzed by TG/DSC. The results revealed that the temperature at which weight gain begins was 495 °C and 545 °C, respectively. The oxidation behaviors of the lead- and bismuth-modified borosilicate glass coatings were compared at 500 °C, and the antioxidation properties of the former were further examined from 500 to 900 °C. The oxidation results indicated that mixing lead glass with borosilicate glass realized effective oxidation resistance in the full temperature range. With a lead content of 20%, the lead–borosilicate glass coating was able to protect C/C substrates from oxidation. The corresponding weight loss of the lead-glass-coated samples was −1.89% when oxidized at 500 °C for 10 h, while the weight loss was −2.55% when further oxidized at 900 °C for 10 h. However, mixing bismuth glass with borosilicate glass was difficult to achieve the oxidation resistance of the coating at 500 °C due to the significant phase separation.

## 1. Introduction

As widely used brake materials for aircraft, C/C composites possess a series of merits such as being lightweight, having a long life, and excellent high-temperature performance, etc. [1,2]. During the braking process of an aircraft, the friction effect between brake discs quickly converts kinetic energy into heat. Under normal braking conditions, the average temperature of the brake disc is generally between 450–800 °C, while the surface temperature of the brake disc can quickly rise to 900 °C when emergency braking occurs. In some extreme cases, the temperature may even rise to 1100 °C [3,4,5]. In air conditions, when the temperature is above 400 °C, C/C will gradually oxidize, and the oxidation becomes intense with rising temperature [6]. To avoid rapid oxidation damage, the non-friction surface of the C/C brake disc is usually protected by an antioxidation coating [7,8]. To provide a long-term antioxidation effect, the self-healing ability of the coatings for C/C composites is usually required [9,10,11,12]. Owing to the frangibility of glass coating, it is probably subjected to damage during the transportation, installation, disassembly, and maintenance of the C/C brake disc [13,14]. The repair of the glass coating, especially for on-site repair, is important in practical applications [15].

Currently, the most common method to prepare the on-site repair coatings is preceramic-precursor-derived ceramic technology [16]. The on-site repair coating is usually formed by adding a polymer precursor to the glass powders and undergoing simple low-temperature curing [17]. In order to achieve oxidation resistance, this preparation method needs a rapid heating rate to form the glass phase coating. Under normal braking conditions, the braking temperature can rise rapidly in a short period of time. With the rapid increase in the surface temperature of the brake discs, the glass particles adhere on the C/C surface and transform into liquid phases, thus forming a glass coating. To complete the conversion of glass powders to glass coating, high temperature and time are required [10,18,19]. Under normal braking conditions, the surface temperature of the brake disc can reach 600 °C in 40 s [18]. In this situation, the glass powder is still in the state of particles, while the C/C substrate has started to oxidize [19]. Therefore, the ability of rapid formation of glass coating is essential for the repair coating.

Aiming at achieving antioxidation of C/C brake discs, we previously prepared an PSN/borosilicate glass-B_4_C coating and realized effective oxidation resistance between 700 and 900 °C [20]. However, borosilicate glass does not start to soften until 650–680 °C, thus, making it difficult for the single borosilicate glass coating to provide oxidation resistance below 650 °C. Therefore, the on-site repair coating with a single softening temperature range cannot suit the anti-oxidation requirements during the temperature rise [21,22]. To achieve a long-term oxidation protection effect for the repair coating in a full-temperature range (500–900 °C), constituents with lower softening temperatures must be contained in the glass coating. For this purpose, lead glass and bismuth glass may be potential choices for mixing with the borosilicate glass, as their softening temperature are 380–400 °C and 450–470 °C, respectively.

In this study, multi-component glass coatings with a wide softening temperature range to suit the antioxidation requirements of C/C brake materials in the full-temperature range were proposed. Two different lead/borosilicate and bismuth/borosilicate glass on-site repair coatings were compared. The glass coating was formed by brushing glass slurry on C/C samples and then heat treating at 200 °C for 0.5 h. Detailed softening behaviors of the glass coatings were analyzed by TG technology. The oxidation behaviors of the prepared repair coating samples were examined under different temperatures. After isothermal oxidation tests, the surface and cross-sectional morphologies were characterized. The effect of the addition of glass with lower softening temperature on the oxidation behaviors was accordingly discussed.

## 2. Materials and Methods

### 2.1. Preparation

**Figure 1:**
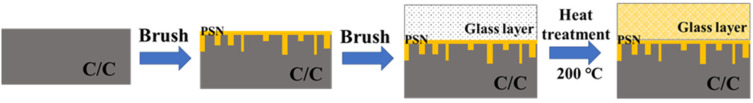
Preparation process of the glass coating on C/C substrate.

The C/C substrate used in this study was cut from 3D needled C/C brake discs. The size was 10 mm × 8 mm × 6 mm and the corresponding density was about 1.7 g/cm^3^. The anti-oxidation glass coating in this study is designed with a dual-layer structure, and its preparation process is shown in Figure 1. Liquid polysilazane (PSN) was chosen as the ceramic transition layer and adhesive agent. The viscosity of the PSN at room temperature was 12–200 mPa∙s. Firstly, the PSN solution diluted by ethanol was directly brushed on the C/C substrate. After brushing the PSN transition layer, the preparation of the glass slurry was initiated. The glass powders with an average size of 5 µm were employed as coating sources, including lead, bismuth, borosilicate glass, and B_4_C ceramic powders. Table 1 lists the basic characteristics of the coating powders [23]. To acquire a lead–borosilicate glass coating, lead glass, borosilicate glass, and B_4_C were added into PSN solution with a weight ratio of 1:9 and were then mixed in a high-energy ball mill at 120 rpm for 4 h to form a slurry. After that, the as-obtained glass slurry was brushed onto the PSN-layer-covered C/C samples. The lead–borosilicate glass coating was finally formed by heat treatment in a furnace at 200 °C for 0.5 h. Similarly, the bismuth–borosilicate glass coating samples were also prepared. The C/C coated with 10 wt.%, 20 wt.%, 30 wt.%, and 40 wt.% of lead and bismuth glass coating are represented by Pb10/Bi10, Pb20/Bi20, Pb30/Bi30, and Pb40/Bi40, respectively.

### 2.2. Tests and Characterization

The thermogravimetric characteristics of borosilicate glass–B_4_C, lead glass–borosilicate glass–B_4_C, and bismuth glass–borosilicate glass–B_4_C were analyzed by thermogravimetry-differential scanning calorimetry (TG/DSC, STA 449C, Netzsch, Selb, Germany) from room temperature to 900 °C under air atmosphere; the heating rate was set as 10 °C/min. The samples used for TG/DSC tests weighed 10–15 mg.

Isothermal oxidation tests were used to examine the oxidation resistance ability of the lead–borosilicate and bismuth–borosilicate glass coated C/C samples. The oxidation temperature was set as 500 °C and the duration was 10 h. To acquire the weight change with the extension of oxidation time, the coating samples were taken out and weighed every two hours during the oxidation tests. Analytical balance (AG135, Mettler Toledo, Switzerland) with a sensitivity of ±0.1 mg was used for measuring the weight variations of the samples. For each weighing, at least five samples were measured. To further analyze the antioxidation mechanism, the lead–borosilicate glass coating samples were oxidized from 500 to 900 °C for 2 h.

The surface and cross-sectional microstructure of the coating samples before and after oxidation were characterized by scanning electron microscopy (SEM, S-4700, Hitachi, Tokyo, Japan). The elemental composition of the coatings was analyzed by energy-dispersive spectroscopy (EDS, EDAX, Warrendale, PA, USA).

## 3. Results and Discussion

### 3.1. Thermal Characteristics of the Glass Powders

**Figure 2:**
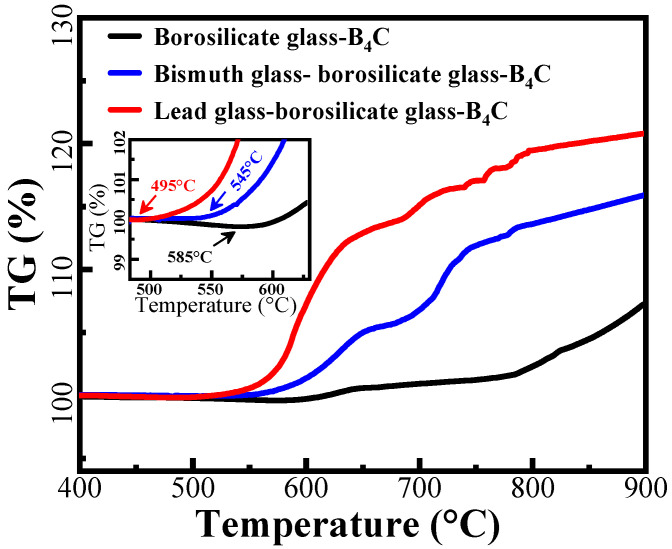
TG curves of different glass mixtures.

Figure 2 shows the TG analysis of different glass mixtures. At 585 °C, single borosilicate–B_4_C glass starts to gain weight. This means that B_4_C particles are starting to oxidize at this temperature as the oxidation of B_4_C is a weight-increasing process. By adding bismuth glass into the powders, the temperature at which weight gain begins is decreased to about 545 °C. By adding lead glass, this temperature is significantly decreased to 495 °C. Therefore, adding glass with a lower softening temperature can change the initial oxidation temperature of borosilicate–B_4_C glass. Between 400 and 500 °C, the lead glass and bismuth glass will partially transform to a liquid glass phase, accelerating the rearrangement of B_4_C particles, and thus increasing the oxidation rate of B_4_C. On the other hand, these TG curves are not observed to sharply decrease when the temperature is close to 900 °C, which indicates that serious volatility of lead glass and bismuth glass did not occur at this temperature. Therefore, borosilicate glass and B_4_C can also inhibit the volatilization of lead and bismuth glasses with lower softening temperatures.

### 3.2. Microstructure before Oxidation

**Figure 3:**
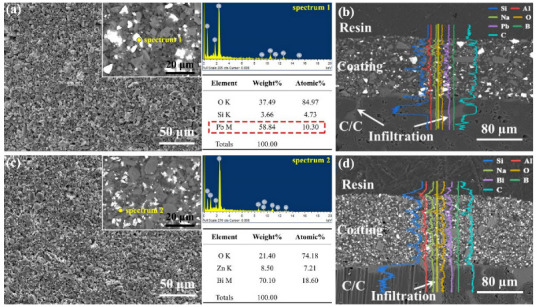
SEM and enlarged backscattered electron (BSE) images of the coating: (**a**) surface of the PSN/lead–borosilicate glass–B_4_C coating and corresponding EDS analysis; (**b**) cross-sectional image and EDS line results of PSN/lead–borosilicate glass–B_4_C coating; (**c**) surface of the PSN/bismuth–borosilicate glass–B_4_C coating and corresponding EDS analysis; and (**d**) cross-sectional image and EDS line results of PSN/bismuth–borosilicate glass–B_4_C coating.

Figure 3 shows the representative microstructure of the PSN/lead–borosilicate glass–B_4_C and PSN/bismuth–borosilicate glass–B_4_C repair coatings after curing at 200 °C for 2 h. It can be seen that the coatings are constructed by several glass particles and the structure of the coatings is uniform and dense. From the corresponding BSE image and EDS analysis, lead and bismuth glass particles are uniformly distributed in the coatings. During the low-temperature curing process, the cross-linking of PSN cures and connects the solid phases of borosilicate glass, B_4_C, and lead (or bismuth) glass to achieve an integrated structure. The cross-sectional morphology (Figure 3e,f) indicates that the thicknesses of the PSN/lead–borosilicate glass–B_4_C and PSN/bismuth–borosilicate glass–B_4_C repair coating is about 100 µm and 120 µm, respectively. According to the elemental line scanning analysis, the transition layer is well infiltrated to the C/C substrate, guaranteeing a good combination of the coating layer and substrate material.

### 3.3. Oxidation Behaviors at 500 °C

**Figure 4:**
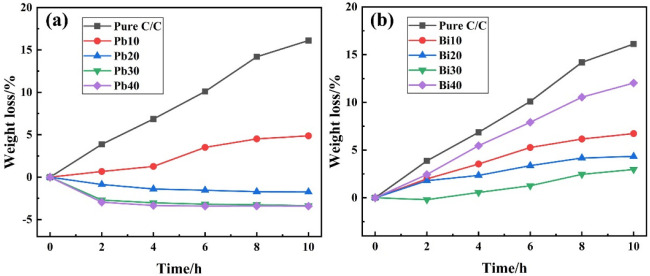
The isothermal oxidation curves of C/C with and without coatings at 500 °C in air: (**a**) PSN/lead–borosilicate glass–B_4_C coatings; and (**b**) PSN/bismuth–borosilicate glass–B_4_C coatings.

After isothermal oxidization at 500 °C for different durations, the weight change of the samples with or without coating layers is measured and summarized in Figure 4. Without coating protection, the weight loss of C/C samples exhibits a maximum value of 23.22% after oxidizing for 10 h. When protected by lead–glass coatings, the weight loss of the samples is significantly reduced, which indicates the improvement of the antioxidation property (as shown in Figure 4a). By increasing the lead glass content from 10% to 40%, the weight variation of the coating samples gradually changes from weight loss to weight gain. When the lead glass content is low, i.e., the Pb10 samples, the weight loss gradually increases with the oxidation, and the maximum weight loss after 10 h oxidation reaches 4.89%. Compared with C/C samples not covered by coatings, the lead-modified glass coating effectively protected C/C composites from oxidation. When lead content increases, i.e., in the Pb20 samples, it shows steady weight gain with the extension of oxidation time. There is no weight loss even continuously oxidized for 10 h. Further, when the lead glass content increases to above 30%, the weight of samples exhibits a rapid gain once the oxidation occurs. After 4 h of oxidation, the weight gain of Pb30 and Pb40 reaches their maximum. Subsequently, the weight of the samples barely changes again. After 10 h oxidation, the weight loss of Pb30 and Pb40 was −3.27% and −3.41%, respectively.

Figure 4b presents the isothermal oxidation curves of bismuth–glass coating samples. Unlike the lead-modified-glass coating samples, the PSN/bismuth–borosilicate glass–B_4_C coated C/C samples always exhibit weight loss proceeding oxidation. When increasing the content of bismuth glass from 10% to 30%, the corresponding weight loss of the coated samples gradually decreases. After oxidizing for 2 h, the weight loss of Bi10 and Bi20 samples are 1.98% and 1.77%, respectively, while the Bi30 samples exhibit a slight weight gain of 0.22%. However, the weight of Bi30 is subsequently lost as the oxidation process continues. After 10 h of oxidation, the weight loss percentages of Bi10, Bi20, and Bi30 are 6.92%, 4.12% and 2.51%, respectively. However, when the bismuth glass content is up to 40%, the weight loss of the samples is more severe. The oxidation tendency is similar with that of pure C/C, and the corresponding oxidation weight loss curve rises almost linearly. After oxidizing for 10 h, the weight loss of Bi40 can rise to as high as 12.01%. Therefore, the introduction of bismuth into the glass coating cannot provide effective protection for C/C composites.

### 3.4. Morphology after Oxidation

#### 3.4.1. PSN/Lead–Borosilicate Glass–B_4_C Coatings

**Figure 5:**
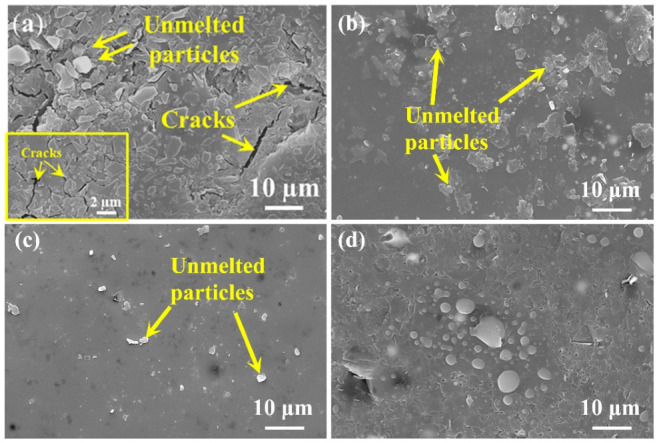
Surface morphologies of the PSN/lead–borosilicate glass–B_4_C coatings with different lead glass content after oxidation at 500 °C for 2 h: (**a**) Pb10; (**b**) Pb20; (**c**) Pb30; and (**d**) Pb40.

The surface microstructure of PSN/lead–borosilicate glass–B_4_C coatings with different lead glass content after oxidation are presented in Figure 5. With the increase in lead glass content, some unmelted borosilicate glass and B_4_C on the coating surface gradually disappear and the surface becomes smoother. For Pb10 and Pb20 coatings shown in Figure 5a,b, some unmelted particles are still wrapped in the glass layer and the surface with liquid flow state and fixed solid morphologies coexist. In addition, there are lots of cracks between the particles in Figure 5a while the liquid glass phase is completely spread out and covers some of the solid particles in Figure 5b. For the coatings with high lead glass content, the whole surface of the samples are covered by a smooth and flat glass layer, as shown in Figure 5c,d. Nearly no solid borosilicate glass powders and B_4_C particles can be found in the coating surface. According to the TG analysis in Figure 2, only lead-modified borosilicate glass–B_4_C powders exhibit weight gain at 500 °C. In this situation, the lead glass can form a liquid glass phase and flow to seal the voids between unmelted particles (borosilicate glass, B_4_C) at 500 °C. By increasing the lead glass content, there are more liquid glass phases in the coatings, resulting in the complete coverage of the borosilicate glass and B_4_C powders.

**Figure 6:**
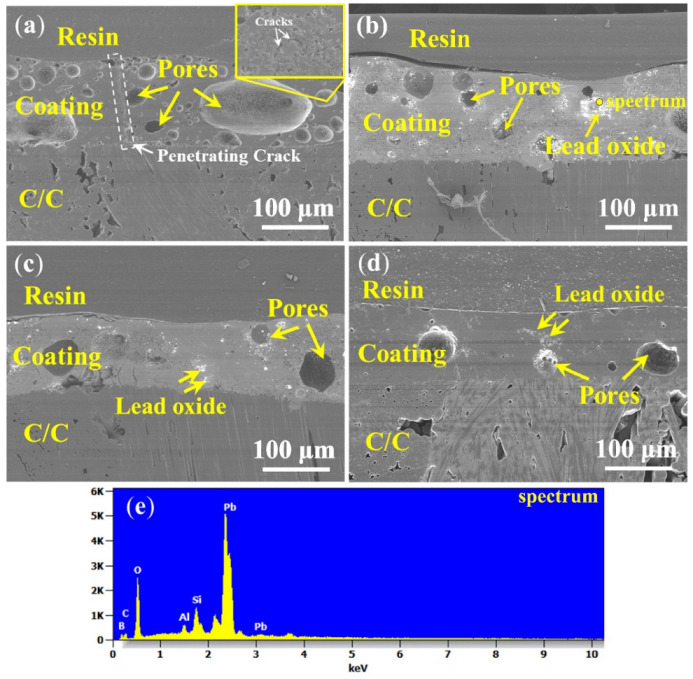
Cross-sectional images of PSN/lead–borosilicate glass–B4C coatings with different lead glass content after 500 °C oxidation for 2 h in air: (**a**) Pb10; (**b**) Pb20; (**c**) Pb30; (**d**) Pb40; and (**e**) EDS result of the point labeled in (**b**).

Figure 6 shows the corresponding cross-section morphologies of the coatings after oxidation. The thickness of the coatings Pb10 and Pb20 are about 110–120 µm, while that of Pb30 and Pb40 are about 100–110 µm, respectively. By increasing the lead glass content, the amount of pores in the coatings after oxidation gradually decreases; relatively more pores existed in the coating Pb10 than in the others. In addition, a penetrating crack is observed in Pb10. Combining the oxidation surface of the coating in Figure 5a and the isothermal oxidation curves in Figure 4a, it can be found that the coating with 10% lead glass cannot protect C/C well from oxidation as defects in the glass materials, such as pores and cracks, are inevitable in the coating. Since the fluidity of the Pb10 coating is not good (as a lot of unmelted particles are found in Figure 5a), the cracks generated by the volume shrinkage cannot be healed on time and oxygen will penetrate through these cracks or gaps to oxidize C/C. As shown in Figure 6b,c, although there are still pores after oxidation, the coatings Pb20 and Pb30 can effectively protect the C/C. The decrease in the amount of pores is due to the increase in the lead glass liquid phase, which results in more particles melting and more coverage (the SEM images of the coating surface in Figure 5b,c) and more B_4_C oxidation (the weight gain in Figure 4). As shown in Figure 6d, as the content of lead glass increases to 40%, the unmelted particles are nearly covered and a comparatively dense and smooth coating is spread on the C/C. In addition to pores, some white spots also existed in the coatings Pb20, Pb30, and Pb40. Combining with the EDS results shown in Figure 6e, these white spots can be determined as lead oxide.

#### 3.4.2. PSN/Bismuth–Borosilicate Glass–B_4_C Coatings

**Figure 7:**
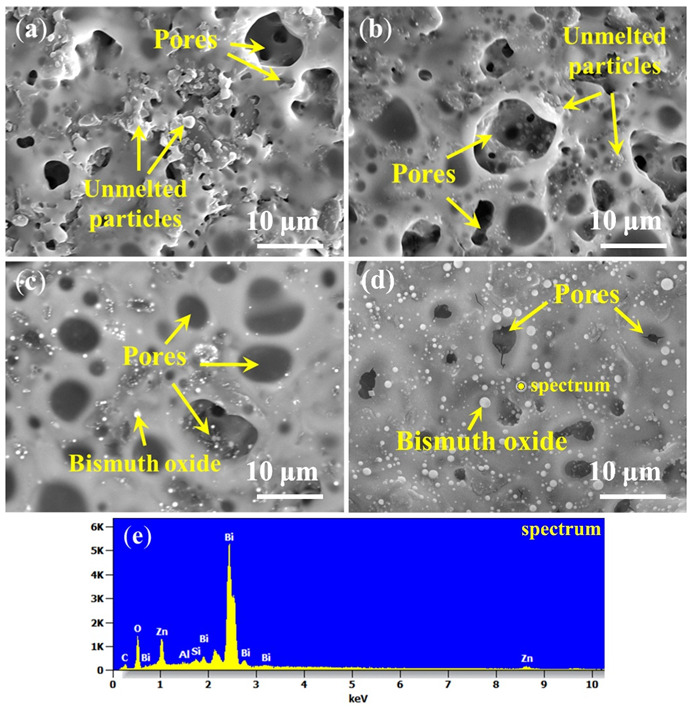
Surface images of the PSN/bismuth–borosilicate glass–B_4_C coatings with different bismuth glass content after oxidation at 500 °C for 2 h in air: (**a**) Bi10; (**b**) Bi20; (**c**) Bi30; (**d**) Bi40; and (**e**) EDS result of point labeled in (**d**).

After oxidation at 500 °C, the coating surface images with different bismuth glass content are presented in Figure 7. It indicates that some unmelted borosilicate glass powders and B_4_C are shown on the surface (Figure 7a,b), and with the increase in bismuth glass content, these solids can be covered by the bismuth glass (Figure 7c,d). Although the borosilicate glass powders and B_4_C can be wrapped in the formed coatings, there are still a certain number of pores that remain unsealed in the repair coating. In other words, these pores inside the coating, which are gradually formed during the softening of the glass particles, cannot be excreted. As shown in Figure 7a,b, most pores on the coating surface are open pores, which will provide oxygen diffusion paths and result in severe oxidation of the C/C substrate. Thus, the coating with low bismuth glass content suffers great damage after oxidation at 500 °C. As shown in Figure 7c, there are a certain amount of closed pores, which can block the entry of oxygen to an extent, so the antioxidation performance of the coating is slightly improved, as shown in the weight loss curves in Figure 4b. On the other hand, Figure 4b also indicates that the weight loss of the coating samples with high bismuth glass content is lower than those coatings with lower bismuth glass content. However, when the bismuth glass content is increased up to 40 wt.%, the pores on the coating surface gradually changed from the original closed pores (Figure 7c) to open pores (Figure 7d). Hence, oxygen will re-enter the C/C substrate through these defects and result in severe weight loss of Bi40 samples. Furthermore, it can be seen in Figure 7 that the bismuth glass is gradually precipitated from the glass coating with the increase in bismuth content; this phenomenon is herein defined as phase separation. The phase separation is caused by the different melting rates of different glasses. The two glasses do not wet each other, causing the first molten glass in the coating to gradually agglomerate into the liquid phase and separate out from the later molten glass. In contrast, the same phenomenon is not significant in the lead-modified glass coatings.

**Figure 8:**
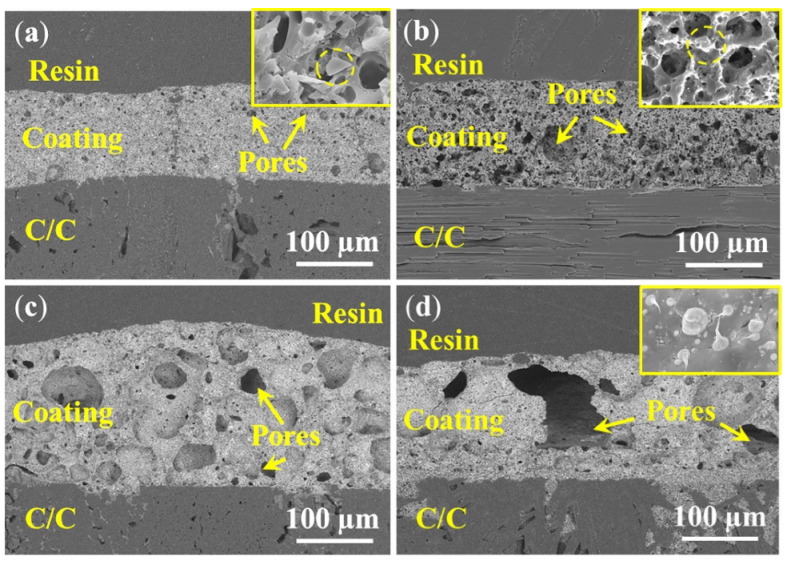
Cross-sectional images of PSN/bismuth–borosilicate glass–B_4_C coatings with different bismuth content after oxidation at 500 °C for 2 h in air: (**a**) Bi10; (**b**) Bi20; (**c**) Bi30; and (**d**) Bi40.

The corresponding cross-sectional morphologies of PSN/bismuth–borosilicate glass–B_4_C coated C/C samples with different bismuth glass content are exhibited in Figure 8. The coating thickness (Figure 8a,b,d) are all about 115 µm, while that in Figure 8c is about 130 µm. As the content of the bismuth glass increases, the size of pores in the coating also increases. The diameter of the pores in the Bi10 samples are approximately 2–4 µm, which is smaller than the diameters of the Bi20 samples of about 8–10 µm. When adding more bismuth glass, a large number of internal holes are generated, causing severe deformation of the coating; as shown in Figure 8c, the diameters of the pores in the Bi30 samples are about 30–40 µm. As shown in Figure 8d, due to the formation of macro pores, whose diameter can exceed one hundred micrometers, disastrous breakage of the Bi40 coating occurs, resulting in continuous oxidation of the C/C substrate. In addition, the enlarged view of the Bi10 and Bi20 samples shown in Figure 8a,b indicates that some unmelted particles exist in the coating layer, and these particles are gradually covered by the liquid glass phase with an increase in bismuth content (seen Figure 8d). The enlarged view in Figure 8d also indicates that bismuth glass separates out from the glass layer, i.e., the phase separation phenomenon of the coating, which is consistent with the morphology observed in Figure 7.

**Figure 9:**
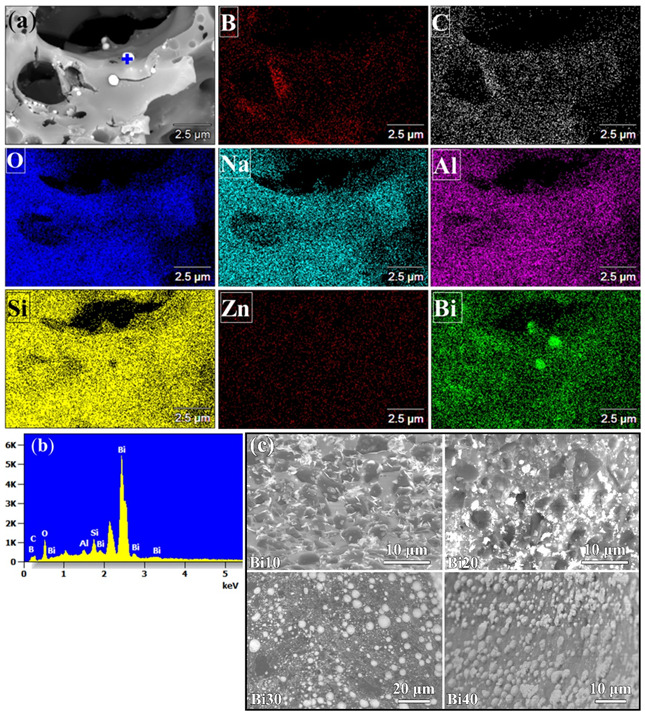
SEM morphologies of phase separation of the PSN/bismuth–borosilicate glass–B_4_C repair coatings: (**a**) elemental maps; (**b**) EDS analysis of (**a**); and (**c**) surface.

Figure 9 characterizes the phase separation of the PSN/bismuth–borosilicate glass–B_4_C coating. It can be seen from the elemental maps in Figure 9a that the phase separation mainly occurs on the pore regions of the surface. The precipitated phase from the glass coating has a main component of Bi_2_O_3_. EDS analysis in Figure 9b also implies that the precipitated glass phase mainly contains the Bi and O element. Moreover, it can be observed from Figure 9c that the phase separation of these bismuth oxides becomes more and more significant as the bismuth glass content increases. The result of phase separation will cause polymerization between the glass with a lower softening point, severe volume shrinkage inside the glass coating, and eventually the formation of fatal pores.

### 3.5. Oxidation Behavior under Different Temperatures

**Figure 10:**
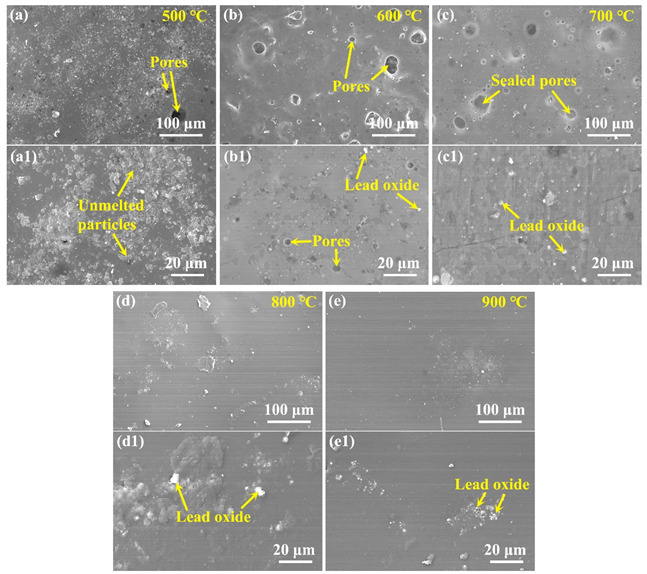
Surface images of the PSN/lead–borosilicate glass–B_4_C coated C/C after oxidation at different temperatures for 2 h: (**a**,**a1**) 500 °C; (**b**,**b1**) 600 °C; (**c**,**c1**) 700 °C; (**d**,**d1**) 800 °C; and (**e**,**e1**) 900 °C.

From above, the PSN/borosilicate glass–B_4_C coatings that contain lead glass can possess an effective anti-oxidation effect at 500 °C while those that contain bismuth glass cannot effectively protect C/C from oxidation in the same situation. To further examine the antioxidation characteristics of lead-modified glass coatings, the anti-oxidation property of Pb20 samples were tested under different temperatures. Table 2 exhibits the weight loss of the samples with the variation of oxidation temperature. It shows that all the coating samples exhibit weight gain when oxidizing at 500–900 °C, and the gain value slightly increases when the oxidation temperature increases. After 2 h of oxidation, the surface morphology evolution with the oxidation temperature is shown in Figure 10. As shown in Figure 10a,a1, when the oxidation temperature is below 600 °C, lead glass provides an important contribution to the oxidation resistance of the coating. Due to the addition of lead glass with a lower softening temperature, it can soften and flow to connect the glass particles as a whole, thus ensuring the oxygen cannot enter the C/C substrate though the gaps. Therefore, though some unmelted particles (borosilicate glass, B_4_C) are found in the coating layer, they are tightly wrapped by the liquid lead glass. Further, from the enlarged view shown in Figure 10a1, the liquid lead glass can fully connect particles to form a dense coating. That is why the weight loss of the coated samples showed a negative value in Table 2. Above 600 °C, the coating surface gets smoother and denser with increasing temperature. As shown in Figure 10b–e, unmelted particles (borosilicate glass, B_4_C) gradually disappear with the increase in oxidation temperature. This is because B_4_C can be rapidly oxidized to form the liquid B_2_O_3_ phase to cover the surface when the temperature is above 600 °C (just as analyzed in Figure 2). When oxidized at 600 °C and 700 °C, although micro pores still exist in the coating, it can still provide effective oxidation resistance for C/C substrates, since these pores are not penetrative pores. When the temperature rises, i.e., at 800 °C and 900 °C, the coating is constructed by a dense and smooth glass layer. The dense glass coating could effectively protect carbon phases in the substrate from oxidation as the oxygen diffusion is significantly inhibited. From the magnified morphology in Figure 10d1,e1, it is found that little lead oxide is unevenly distributed on the coating surface, however, it does not infect the integrity of the coatings.

**Figure 11:**
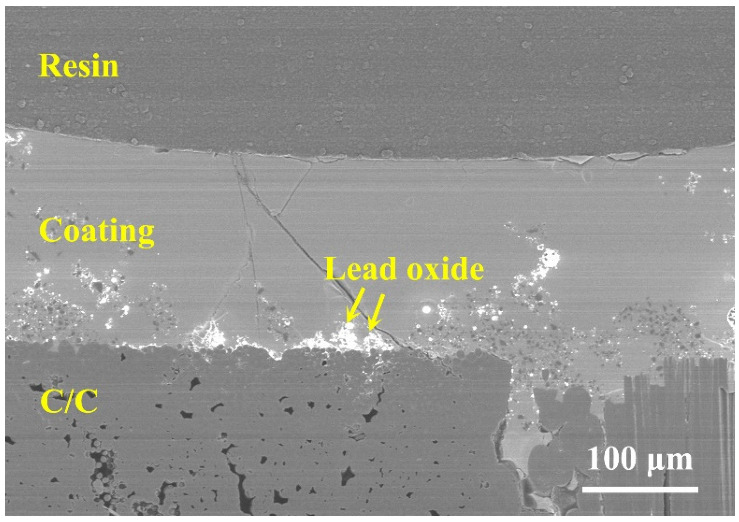
Cross-sectional morphology of PSN/lead–borosilicate glass–B_4_C coated C/C with Pb20 at 900 °C for 10 h in air.

To further examine the anti-property of the lead glass coating, the samples were further oxidized at 900 °C for 10 h. The corresponding weight loss of the samples is −2.55%, which indicates that even with the extension of oxidation duration, the effective anti-oxidation ability of the lead glass coating is still working. The corresponding cross-section morphology after 10 h of oxidation is shown in Figure 11. A smooth and dense coating layer is covered on C/C substrate, indicating a good long-term antioxidation property of the PSN/lead–borosilicate glass–B_4_C at high temperature. Under such a long time and a high-temperature oxidation condition, borosilicate glass in the coating could fully soften and flow on the surface and cracks or other defects generated during the oxidation could be healed in time. Meanwhile, liquid B_2_O_3_ could also flow and heal those defects. Combining with the TG analysis in Figure 2, no severe volatilization will occur at 900 °C for this coating material. Above 650 °C, borosilicate glass with higher softening temperature and dispersively distributed B_4_C particles can inhibit the volatility of lead glass with low softening temperature. Therefore, the coating layer remained intact and smooth even after oxidation for 10 h. The results indicate that mixing lead glass and borosilicate glass to form the coating provided effective oxidation resistance for C/C composites in the full temperature range (500–900 °C).

## 4. Conclusions

In this work, on-site repair glass coating with multi-components was proposed to fulfil the demand of continuous antioxidation effect for C/C brake materials in applications. Aiming at applications requiring the full temperature range (500–900 °C), i.e., the true service temperature range, lead glass and bismuth glass with lower softening temperature were introduced into borosilicate glass–B_4_C powders to prepare the coating materials. The oxidation behaviors of lead- and bismuth-modified borosilicate glass repair coatings were compared. The results indicated that mixing lead glass with borosilicate glass can obtain coating materials with excellent antioxidation properties in the full temperature range. Introducing lead glass decreased the softening temperature of the coating material, whose softening temperature was below 500 °C, which guaranteed the effective antioxidation property when the temperature is below 600 °C. The coating containing 20% lead glass is enough to provide excellent oxidation resistance, which can protect the C/C from oxidation and the corresponding weight loss at 500 °C and 900 °C for 10 h were −3.41% and −2.55%, respectively. Meanwhile, borosilicate glass in the coating can inhibit the volatility of lead glass, which effectively prevents C/C substrate from being oxidized at 650–900 °C. Therefore, lead/borosilicate glass coating possessed excellent oxidation resistance in the full temperature range. However, when introducing bismuth glass with softening temperature of 450–470 °C into the borosilicate glass, due to the large amount of phase separation during the oxidation process, the coating experienced severe volume shrinkage, resulting in the formation of a large number of pores in the coating. It is difficult to provide effective oxidation resistance for C/C composites at low temperature. In summary, the lead-modified borosilicate glass–B_4_C coating with 20% lead glass is more favorable for practical applications. Owing to its anti-oxidation property in the full temperature range, the repair coating can be directly brushed on the exposed C/C substrate and cured to guarantee the integrity of the glass coating and provide an effective antioxidation effect.

## Figures and Tables

**Table 1:** The basic characteristics of glass powders.

Glass Powders	Main Components	Average Size (μm)	Softening Temperature (°C)
Lead glass	SiO_2_, B_2_O_3_, PbO	3	380–400
Bismuth glass	BiO_2_, B_2_O_3_, ZnO	3	450–470
Borosilicate glass	SiO_2_, B_2_O_3_, Na_2_O, Al_2_O_3_	5	650–680

**Table 2:** Weight loss of the Pb20 samples after being oxidized at different temperatures for 2 h.

	500 °C	600 °C	700 °C	800 °C	900 °C
Weight loss (%)	−0.94	−1.51	−1.29	−2.22	−2.60

## Data Availability

The data presented in this study are available on request from the corresponding authors.

## References

[1] Fan S.W., Zhang L.T., Xu Y.D., Cheng L.F., Lou J.J., Zhang J.Z., Yu L. (2007). Microstructure and properties of 3D needle-punched carbon/silicon carbide brake materials. Compos. Sci. Technol..

[2] Xiong X., Huang B.Y., Li J.H., Xu H.J. (2006). Friction behaviors of carbon/carbon composites with different pyrolytic carbon textures. Carbon.

[3] Choi J.H., Lee I. (2004). Finite element analysis of transient thermoelastic behaviors in disk brakes. Wear.

[4] Zhang C.Q., Zhang L.T., Zeng Q.F., Fan S.Q., Cheng L.F. (2011). Simulated three-dimensional transient temperature field during aircraft braking for C/SiC composite brake disc. Mater. Des..

[5] Zhang J., Xia C.G. (2013). Research of the transient temperature tield and friction properties on disc brakes. Adv. Mat. Res..

[6] Shemet V.Z., Pomytkin A.P., Neshpor V.S. (1993). High-temperature oxidation behaviour of carbon materials in air. Carbon.

[7] Schulte-Fischedick J., Schmidt J., Tamme R., Kröner U., Arnold J., Zeiffer B. (2004). Oxidation behaviour of C/C–SiC coated with SiC–B_4_C–SiC–cordierite oxidation protection system. Mater. Sci. Eng. A.

[8] Fu Q.G., Li H.J., Shi X.H., Li K.Z., Wang C., Huang M. (2006). Double-layer oxidation protective SiC/glass coatings for carbon/carbon composites. Surf. Coat. Technol..

[9] Feng T., Li H., Shi X., Yang X., Wang S., He Z. (2013). Multi-layer CVD-SiC/MoSi_2_–CrSi_2_–Si/B-modified SiC oxidation protective coating for carbon/carbon composites. Vacuum.

[10] Tian C.Y., Liu H.L. (2010). Fabrication of self-healing ceramic coatings against oxidation for carbon/carbon composites using polysilazane and B_4_C filler. Key Eng. Mater..

[11] Huang J.F., Wang B., Li H.J., Liu M., Cao L.Y., Yao C.Y. (2011). A MoSi_2_/SiC oxidation protective coating for carbon/carbon composites. Corros. Sci..

[12] Fu Q., Zou X., Chu Y., Li H., Zou J., Gu C. (2012). A multilayer MoSi_2_–SiC–B coating to protect SiC-coated carbon/carbon composites against oxidation. Vacuum.

[13] Jacobson N.S., Curry D.M. (2006). Oxidation microstructure studies of reinforced carbon/carbon. Carbon.

[14] Jacobson N.S., Roth D.J., Rauser R.W., Cawley J.D., Curry D.M. (2008). Oxidation through coating cracks of SiC-protected carbon/carbon. Surf. Coat. Technol..

[15] Ozcan S., Filip P. (2013). Wear of carbon fiber reinforced carbon matrix composites: Study of abrasive, oxidative wear and influence of humidity. Carbon.

[16] Fan S.W., Ma X., Li Z., Hu J., Xie Z., Deng J.L., Zhang L.T., Cheng L.F. (2018). Design and optimization of oxidation resistant coating for C/C aircraft brake materials. Ceram. Int..

[17] Lin Y.C., Ruiz E.M., Rateick R.G., McGinn P.J., Mukasyan A.S. (2012). One-step synthesis of a multi-functional anti-oxidation protective layer on the surface of carbon/carbon composites. Carbon.

[18] Yang X., Wang J.X., Fan J.C. (2009). Simulation study of temperature field and stress field of disc brake based on direct coupling method. Mater. Sci. Forum.

[19] Isola C., Appendino P., Bosco F., Ferraris M., Salvo M. (1998). Protective glass coating for carbon-carbon composites. Carbon.

[20] Deng J.L., Hu K.Y., Lu B.F., Zheng B.H., Fan S.W., Zhang L.T., Cheng L.F. (2019). Influence of B_4_C on oxidation resistance of PSN/borosilicate glass-B_4_C field-based repair coating of C/C aircraft brake materials at 700–900 °C. Ceram. Int..

[21] Zhang Y.L., Li H.J., Hu Z.X., Ren J.C., Li K.Z. (2013). Microstructure and oxidation resistance of Si–Mo–B coating for C/SiC coated carbon/carbon composites. Corros. Sci..

[22] Wang K.W., Luo L., Lu Y.H., Yang J., Wang Y.G. (2015). In-field reparation of the damaged coatings for C/C composites. Ceram. Int..

[23] Wang Y., Yang J., Liu J., Fan S., Cheng L.F. (2012). Fabrication of oxidation protective coatings on C/C–SiC brake materials at room temperature. Surf. Coat. Technol..

